# Evaluation of left ventricular systolic function in patients with systemic lupus erythematosus using ultrasonic layer-specific strain technology and its association with cardiovascular events: a long-term follow-up study

**DOI:** 10.1186/s12947-022-00295-0

**Published:** 2022-10-07

**Authors:** Hebin Zhang, Cunxin Yang, Feng Gao, Shanting Hu, Hui Ma

**Affiliations:** Department of Ultrasound, the Affiliated Hospital of Hangzhou Normal University, Zhejiang Province, Hangzhou, 310015 China

**Keywords:** Systemic lupus erythematosus, Left ventricle function, Cardiovascular outcome, Myocardium, Speckle-tracking, Layer-specific strain

## Abstract

**Background:**

Systemic lupus erythematosus (SLE) is a multisystem, autoimmune disease with potential cardiovascular involvement. Layer-specific strain (LSS) analysis is a new method that allows early detection of subtle left ventricular (LV) systolic dysfunction. The aim of this study was to evaluate LV systolic function in patients with SLE using conventional echocardiographic measurements and longitudinal strain (LS) and circumferential strain (CS) by LSS. Furthermore, the association between echocardiographic parameters and the occurrence of cardiovascular events was assessed.

**Methods:**

A total of 162 patients with SLE (the SLE group) who underwent a dedicated multidisciplinary assessment, including echocardiography, were analyzed at the time of their first visits. The control group consisted of 68 age- and sex-matched healthy subjects. LS and CS on endocardial, mid-myocardial, and epicardial layers at 17 cardiac segments were measured. Transmural strain gradient was calculated as the differences in systolic strain between the endocardial and epicardial layers.

**Results:**

Compared with control subjects, patients with SLE had significantly lower LV ejection fraction, LS, and CS values in all layers (*P* < 0.05); LV LS and CS gradient were all lower than control subjects (*P* < 0.05). During a median follow-up period of 83 months (interquartile range: 64–95 months), 59 patients (36.4%) developed cardiovascular events. Using multivariate Cox regression analysis, we found that LV endocardial LS (hazard ratio, 1.014; 95% CI, 1.002–1.035; *P* = 0.025) and CS (hazard ratio, 1.051; 95% CI, 1.027–1.077; *P* < 0.001) demonstrated independent associations with cardiovascular events; whereas LV ejection fraction was not significantly associated with cardiovascular events. The Kaplan–Meier survival curves showed that patients with SLE with lower LV endocardial LS and CS (based on the cutoff values of -21.5% and -29.0%, respectively) experienced higher cumulative rates of cardiovascular events compared with those with higher LV endocardial LS and CS.

**Conclusions:**

In patients with SLE, LV systolic function measured by LV endocardial LS and CS were significantly lower than that of the control group and were associated with cardiovascular events, potentially representing a new technology to improve risk stratification in these patients

## Background

Systemic lupus erythematosus (SLE), which usually occurs in young females, is associated with high cardiovascular morbidity and mortality [1, 2]. Cardiovascular system involvement is common in SLE and can occur in up to 70% of patients with SLE at autopsy [3]. Diagnosis of myocardial involvement, particularly at an early stage, is difficult because SLE may be present without symptoms, and the signs in patients with SLE are often nonspecific. Echocardiography is a convenient and effective tool to assess cardiac structure and function alterations that have been shown to predict clinical outcomes in cardiovascular diseases (CVD) [4]. However, conventional echocardiography is limited by its low sensitivity in detecting myocardial dysfunction and may lead to the underestimation of myocardial involvement in patients with SLE. The use of speckle-tracking.

echocardiography is currently proposed as a more sensitive approach to detecting subclinical myocardial dysfunction than conventional echocardiography parameters such as left ventricular ejection fraction (LVEF) and fractional shorting [5]. Using this advanced echocardiography technique, left ventricular (LV) longitudinal strain (LS) and circumferential strain (CS) have been shown to be clinically valuable for the detection of myocardial dysfunction and risk stratification in several CVDs, including myocardial involvement in autoimmune diseases such as SLE and systemic sclerosis [6, 7], can be measured. Layer-specific strain (LSS) using speckle-tracking methodology has the advantage of quantifying LV LS and CS of the myocardium in three separate layers: endocardial, mid-myocardial, and epicardial which makes it more sensitive and accurate than speckle-tracking echocardiography [8]. However, few studies have assessed the diagnostic value of LV LS and CS for each layer in patients with SLE [9], and to the best of our knowledge, no study has been published on the potential prognostic value of these parameters. The purpose of this study was to assess LV systolic function in patients with SLE using standard echocardiographic and LSS technologic measurements, including values of LS and CS for each layer; in particular, the association between LV LS and CS and the development of cardiovascular events was explored.

## Methods

### Study population

Patients were consecutively recruited from the Department of Rheumatology at the Affiliated Hospital of Hangzhou Normal University for an extensive multidisciplinary from January 2010 and October 2020 and underwent echocardiographic analysis. All patients fulfilled the 2012 American College of Rheumatology (ACR)/Systemic Lupus International Collaborating Clinics (SLICC) classification criteria for SLE [10]. The earliest accessible echocardiographic examination performed during the multidisciplinary assessment was analyzed. The indication to perform echocardiography was the following: (1) potential cardiovascular symptoms (chest pain, dyspnea, syncope, and palpitations); (2) suspected valvular disease and endocarditis (cardiac murmur and other signs of valvular disease and endocarditis); (3) assessment of the cardiac source of embolism in case of recent cerebrovascular accident. Patients diagnosed with pericarditis, valvular disease, conduction system disease, and endocarditis confirmed by baseline echocardiography, acute coronary symptoms or symptomatic heart failure, and previous cardiac surgery were excluded. Before they were included in the multidisciplinary assessment, all patients were given informed consent to use clinically collected data.

The control group consisted of 68 age- and sex-matched healthy subjects who were identified from the echocardiography database with normal heart structure. The study was approved by the institutional ethics committee.

### Clinical data

Demographic and clinical data, including age, sex, height, weight, heart rate, systolic and diastolic blood pressures, SLE characteristics, duration of SLE, comorbidities, and CVD-related risk factors such as smoking, hypertension, diabetes mellitus, dyslipidemia, obesity, and a family history of premature coronary artery disease, were recorded. Specific disease-related characteristics at baseline echocardiographic examination were also reported, including SLE disease activity index 2000 (SLEDAI-2000), positive antinuclear antibody, anti-double-stranded deoxyribonucleic acid, extractable nuclear antigen, antiphospholipid antibodies, reduced creatinine clearance, and elevated C-reactive protein. Patients were followed from the baseline examination of CVD, which was defined as coronary artery disease requiring revascularization, cerebrovascular accident, hospitalization for heart failure, pulmonary embolism, aortic aneurysm and dissection, and development of sustained ventricular or supraventricular arrhythmias. The time till cardiovascular events was calculated from the date of the baseline echocardiographic examination.

### Echocardiography

Comprehensive transthoracic echocardiography was performed using an IE33 or Epiq 7C (Philips Healthcare, Andover, MA, USA; S5-1 transducer) ultrasound system. Examinations were performed with subjects in the left lateral recumbent position. Using the modified biplane Simpson’s formula, LVEF was calculated from measurements of end-diastolic volume (EDV), end-systolic volume (ESV), and stroke volume (SV). Interventricular septum diastolic (IVSd), LV end-diastolic diameter (LVEDd), and posterior wall thickness (PW) were measured from the parasternal long axis using 2D imaging. Relative diastolic wall thickness (RWT) was calculated as the ratio between the sum of IVSd and PW and the LVEDd. LV mass (LVM) was calculated using the formula described by Lang et al. [11]. Left atrial volume index (LAVI) was calculated using the Simpson’s method and indexed to body surface area. Early-diastolic velocity (E) was measured from the mitral inflow profile in the apical four-chamber view. Doppler tissue imaging analysis was performed by placing the sample volume at the septal and lateral mitral annulus, obtaining early-diastolic velocities, the average values(e’), and the E/e’ ratio. LV diastolic dysfunction was registered when it met more than half of the following criteria: annular septal early-diastolic velocity < 7 cm/sec, annular lateral early-diastolic velocity < 10 cm/sec, average E/e’ ratio > 14, left atrial maximum volume index > 34 mL/m^2^, and peak tricuspid regurgitation velocity > 2.8 m/sec [12]. Systolic pulmonary artery pressure (sPAP) was estimated from the peak velocity of the tricuspid regurgitation jet by continuous flow Doppler, and the systolic right atrial pressure was estimated by the inferior vena cava diameter and the degree of respiratory [13].

### Myocardial speckle-tracking

**Fig. 1:**
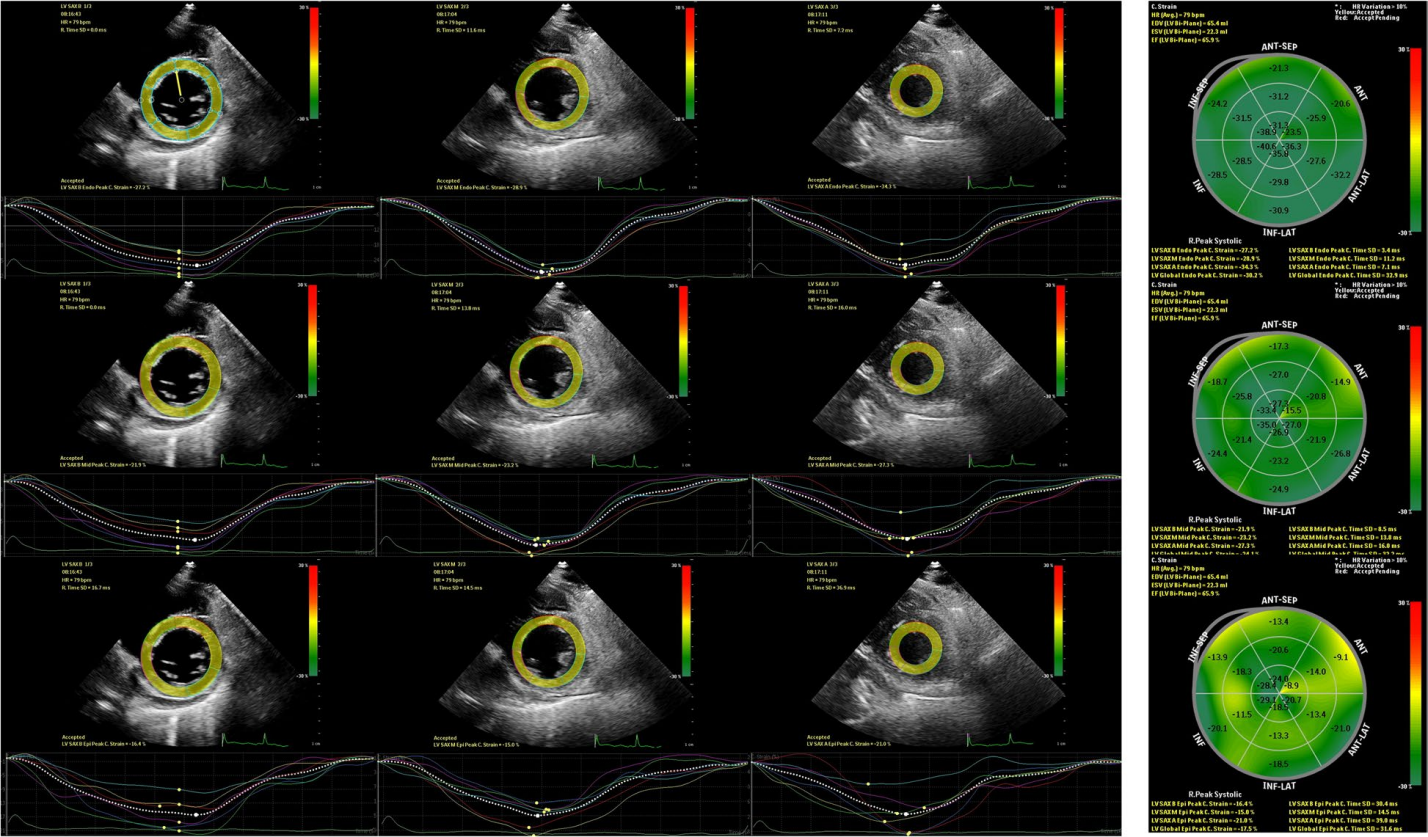
Layer-specific strain curves measurement by speckle-tracking echocardiography from a patient with SLE. LV endocardial CS (**A**), mid-myocardial CS (**B**), and epicardial CS (**C**) were measured from the basal, middle, and apical short-axis dynamic images

After a standard echocardiographic study, the apical long-axis, four- and two-chamber views, and the basal, middle, and apical short-axis planes were scanned using a high frame rate (at least 50 frames/sec) in routine grayscale B-mode for speckle-tracking imaging. These images were stored digitally. All images were obtained while the subjects held their breaths and were stored in a cine loop format for four consecutive beats. The software automatically tracked the myocardial motion during the cardiac cycle. Myocardial tracking echocardiographic was performed using a Qlab multiparameter analysis workstation (version 10.8, Philips Healthcare). The apical long-axis, four-, and two-chamber heart images were analyzed to obtain the peak endocardial, mid-myocardial, and epicardial LS of 17 segments and their corresponding curves, the bull’s eye diagram. The basal, middle, and apical short-axis dynamic images of the LV were traced and used to calculate the LV three-layer CS (Fig. 1). Transmural strain gradient was calculated as the differences of LS and CS between the endocardial and epicardial layers. Myocardial tracking echocardiographic data analyses were conducted by two investigators who had experience with LSS and were blind to the clinical data.

### Statistical analysis

Statistical analyses were performed using SPSS, version 22.0 (IBM, Chicago, IL, USA). Continuous variables are described by mean ± standard deviation for normally distributed variables, and median with interquartile range for non-normally distributed variables, between two groups were compared using *t* test for normally distributed variables and Mann–Whitney test for skewed variables. Categorical variables were described as counts and percentages and were compared using the Pearson chi-square test. The association between LV three-layer LS and CS and cardiovascular events was evaluated using uni- and multivariate Cox regression analyses. Hazard ratios (HR) with 95% confidence interval (CI) are presented. Receiver operating characteristic (ROC) curve analysis was also performed to predict cardiovascular events in patients with SLE. The Kaplan–Meier survival method was used to analyze the cumulative event rates between two groups of patients divided by the cutoff values of LV three-layer LS and CS. Inter- and intra-observer reproducibility was assessed using intraclass correlation coefficients (ICC). All statistical analyses were 2-tailed; *P* < 0.05 was considered to indicate a statistical significance.

## Results

### Baseline clinical characteristics

A total of 168 patients with SLE and 68 healthy subjects were initially included in this study, but six patients with SLE were later excluded because of poor endocardial definition. Clinical characteristics of the overall population are presented in Tables 1 and 2. The SLE and control groups were similar in gender, height, weight, body mass index, and body surface area. Systolic and diastolic blood pressures were significantly higher in the SLE group compared with the control group (*P* < 0.05). Mild disease activity was found in 38.2% (*n* = 62) of patients with SLE, while 34.0% (*n* = 55) had moderate disease activity, and 27.8% (*n* = 45) had high disease activity. The prevalence of comorbidities for patients with SLE were: 30.2% (*n* = 49) had hypertension, 22.8% (*n* = 37) had hypercholesterolemia, 4.3% (*n* = 7) had diabetes mellitus, and 7.4% (*n* = 12) had a family history of premature coronary artery disease. Antiphospholipid antibodies were observed in 28 patients (17.3%) and anti-double-stranded deoxyribonucleic acid in 68 patients (42.0%).

**Table 1:** Baseline characteristics of the study population

Variable	SLE group (*n* = 162)	Healthy controls (*n* = 68)	*P*-value
Age (years)	44.1 ± 16.2	43.9 ± 14.5	0.952
Sex, female (%)	150 (92.6)	61 (89.7)	0.340
Height (cm)	161.7 ± 6.2	162.8 ± 5.7	0.223
Body weight (kg)	59.4 ± 6.3	60.5 ± 5.9	0.227
Body mass index (kg/m^2^)	22.5 (21.9, 23.4)	22.6 (22.1, 23.1)	0.304
Body surface area (m^2^)	1.5 ± 0.1	1.6 ± 0.1	0.192
Heart rate (bpm)	71.4 ± 9.0	69.6 ± 9.1	0.194
Systolic blood pressure (mm Hg)	128.4 ± 17.3	119.9 ± 10.1	** < 0.001**
Diastolic blood pressure (mm Hg)	77.0 (69.0, 90.0)	75.0 (69.0, 79.0)	**0.025**

***NOTE:*** Values are expressed as mean ± standard deviation, number (percentage), or median (interquartile range)

Bold indicates statistically significant values

**Table 2:** Baseline clinical characteristics of patients with SLE

Clinical characteristics	Value
*SLE-related factors*	
SLEDAI-2000 score	9.0 (4.0, 16.0)
Positive antinuclear antibody (%)	162 (100.0)
Positive anti-double-stranded DNA (%)	68 (42.0)
Positive anti-Smith antibody (%)	63(38.9)
Positive U1-small nuclear ribonucleoprotein (%)	38 (23.4)
Positive antiphospholipid antibodies (%)	28 (17.3)
C-reactive protein (mg/L)	16 ± 26
Creatinine clearance (mL/min)	57 ± 34
*Cardiovascular disease-related factors*	
Hypertension (%)	49 (30.2)
Hypercholesterolemia (%)	37 (22.8)
Diabetes mellitus (%)	7 (4.3)
Had a family history of premature coronary artery disease (%)	12 (7.4)
Smoking (%)	6 (3.7)
Body mass index > 25 kg/m^2^ (%)	8 (4.9)
*Medications*	
Hydroxychloroquine (%)	95 (58.6)
Glucocorticoids (%)	74 (45.6)
Immunosuppressive drugs (%)	27 (16.7)
Biological agents (%)	18 (11.1)
*Cardiovascular medications*	
ACE inhibitors/ARBs (%)	30 (18.5)
Calcium channel blockers (%)	21 (12.9)
β-blockers (%)	11 (6.8)
Diuretics (%)	9 (5.6)

***NOTE:*** SLEDAI-2000 score, SLE disease activity index-2000 score; *ACE* angiotensin-converting enzyme, *ARB* angiotensin receptor blocker

Values are expressed as mean ± standard deviation, number (percentage), or median (interquartile range)

### Baseline echocardiographic characteristics

**Fig. 2:**
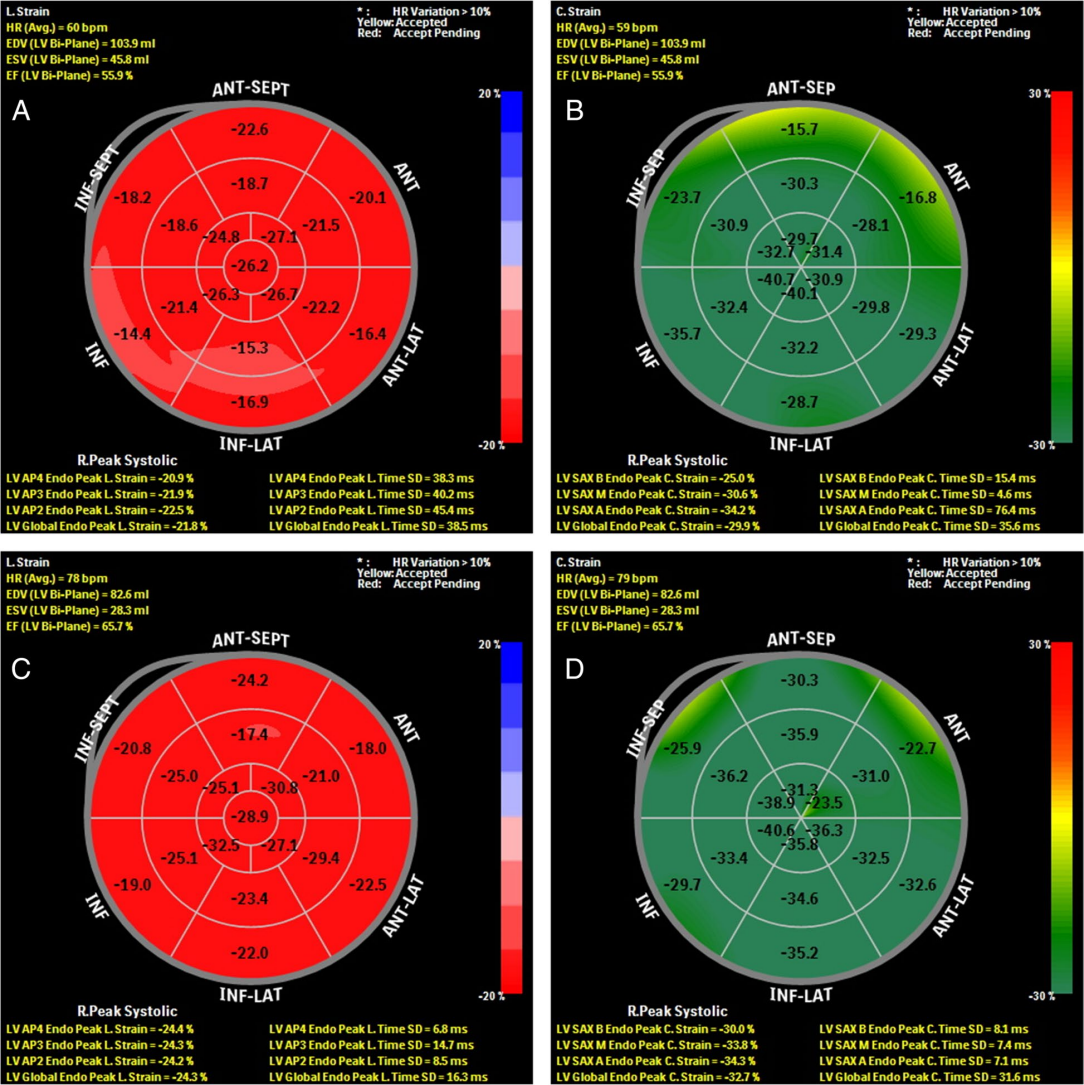
Example of assessment of LV endocardial LS and CS by speckle-tracking echocardiography in patients with SLE (**A**: LV endocardial LS = -21.8%, **B**: LV endocardial CS = -29.9%) compared with healthy control (**C**: LV endocardial LS = -24.3%, **D**; LV endocardial CS = -32.7%) individual displayed with red-blue-, and green–red-coded bull’s-eye plots for LS and CS

**Fig. 3:**
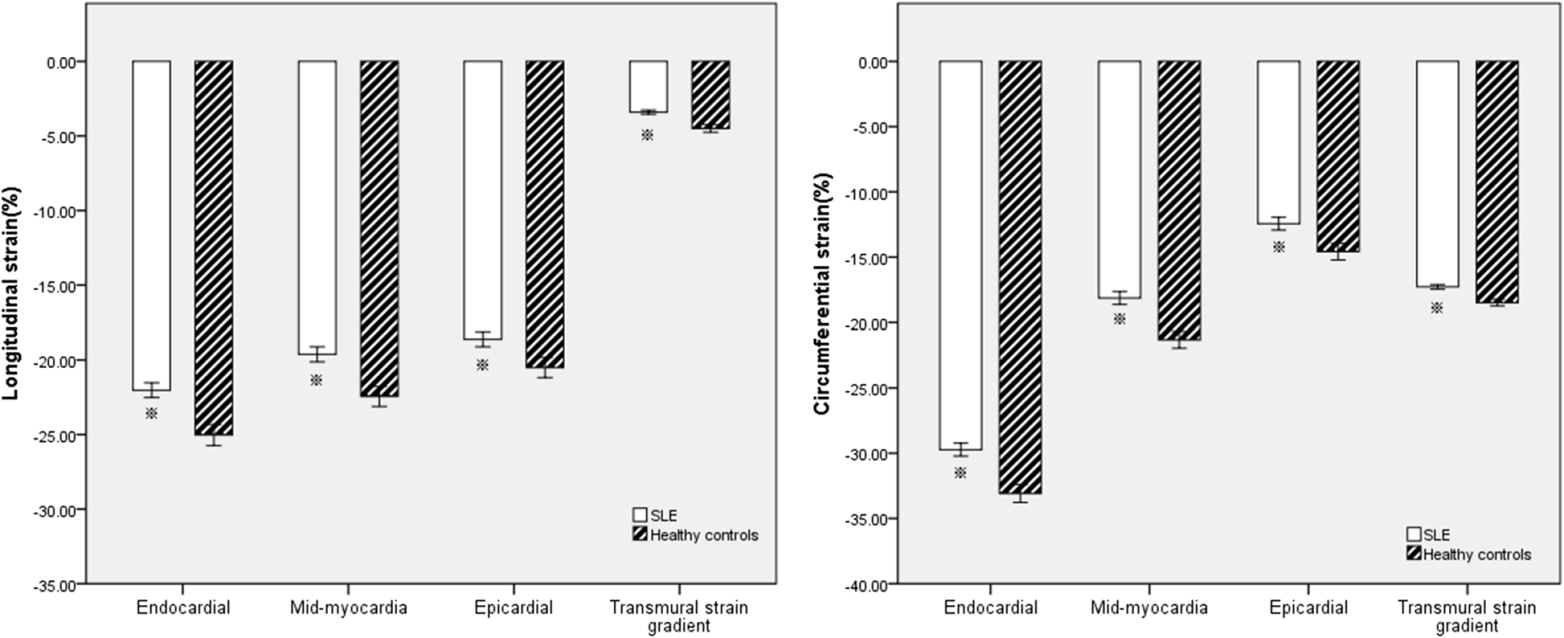
Bar graphs showing LV LS and CS of endocardial, mid-myocardial, epicardial, and transmural strain gradient by LSS analysis in patients with SLE and healthy controls. Data are expressed as mean ± standard deviation values. *P < 0.001 vs. healthy controls

Baseline echocardiographic characteristics of the overall population are presented in Table 3. Compared to healthy subjects, patients with SLE showed significantly lower LVEF (60.6 ± 4.4% vs. 62.6 ± 2.4%, *P* = 0.007), whereas IVSd, LVEDd, PW, EDV, ESV, SV, RWT, and LVM in patients with SLE were significantly higher than that of healthy control subjects. The LV diastolic function, E, e’, and E/e’ were significantly lower in patients compared with healthy controls, and sPAP was significantly higher in patients with SLE, but the prevalence of LV diastolic dysfunction was only seven patients (4.3%) in the overall population. In addition, the LV LS and CS of different myocardial layers in patients with SLE and healthy controls are shown in Table 3, Figs. 2, and 3. LSS measurements were all lower in patients with SLE compared with healthy controls (endocardial LS: -22.0 ± 3.4 vs. -24.8 ± 2.9, *P* < 0.001; LS gradient: -3.4 ± 0.9 vs. -4.5 ± 1.0, *P* < 0.001; endocardial CS: -29.7 ± 3.7 vs. -33.1 ± 3.1, *P* < 0.001; CS gradient: -17.3 ± 1.1 vs. -18.5 ± 1.1, *P* < 0.001).

**Table 3:** Baseline echocardiographic characteristics of patients with SLE compared with healthy control subjects

Variable	SLE group (*n* = 162)	Healthy controls (*n* = 68)	*P*-value
IVSd (mm)	8.4 ± 1.3	7.6 ± 1.1	** < 0.001**
LVEDd (mm)	44.5 ± 4.6	41.8 ± 3.8	** < 0.001**
PW (mm)	7.4 ± 1.0	6.8 ± 1.0	** < 0.001**
EDV (ml)	87.1 ± 18.3	79.5 ± 15.5	**0.003**
ESV (ml)	34.1 ± 8.8	29.9 ± 6.9	**0.001**
SV (ml)	53.0 ± 10.9	50.2 ± 9.0	**0.026**
LVEF (%)	60.6 ± 4.4	62.6 ± 2.4	**0.007**
RWT	0.36 ± 0.04	0.34 ± 0.04	0.056
LVM (g)	108.9 (86.3, 133.1)	88.4 (65.1, 101.8)	** < 0.001**
LVMI (g/m^2^)	68.4 (54.2, 84.1)	54.4 (42.5, 64.2)	** < 0.001**
LAVI (ml/m^2^)	26.0 ± 5.5	24.1 ± 4.7	0.086
E (cm/s)	79.5 (68.6, 92.5)	101.4 (87.6, 105.5)	** < 0.001**
e′ (cm/s)	12.2 ± 1.8	13.1 ± 1.5	** < 0.001**
E/e′	6.6 ± 1.1	7.2 ± 1.4	**0.001**
sPAP (mm Hg)	30.0 ± 8.7	24.5 ± 5.8	** < 0.001**
LV diastolic dysfunction (%)	7 (4.3)	0 (0)	0.072
Endocardial LS (%)	-22.0 ± 3.4	-24.8 ± 2.9	** < 0.001**
Mid-myocardial LS (%)	-19.6 ± 3.1	-22.4 ± 2.7	** < 0.001**
Epicardial LS (%)	-18.6 ± 2.8	-20.5 ± 2.5	** < 0.001**
LS gradient (%)	-3.4 ± 0.9	-4.5 ± 1.0	** < 0.001**
Endocardial CS (%)	-29.7 ± 3.7	-33.1 ± 3.1	** < 0.001**
Mid-myocardial CS (%)	-18.1 ± 3.2	-21.31 ± 2.7	** < 0.001**
Epicardial CS (%)	-12.4 ± 3.0	-14.6 ± 2.6	** < 0.001**
GCS gradient (%)	-17.3 ± 1.1	-18.5 ± 1.1	** < 0.001**

***NOTE:**** IVSd* interventricular septum diastolic, *LVEDd* left ventricular end-diastolic diameter *PW* posterior wall thickness, *EDV* end-diastolic volume, *ESV* end-systolic volume, *SV* stroke volume, *LVEF* left ventricular ejection fraction, *RWT* relative wall thickness, *LVM* left ventricular mass, *LVMI* left ventricular mass index, *LAVI* left atrial volume index, *E* early-diastolic velocity; e′, the average values of early-diastolic velocities at the septal and lateral mitral annulus; E/e′, E/e′ ratio; *sPAP* systolic pulmonary artery pressure, *LS* longitudinal strain, *CS* circumferential strain

Values are expressed as mean ± standard deviation as median (interquartile range)

Bold indicates statistically significant values

### Associations between baseline echocardiographic characteristics and cardiovascular events

During a median follow-up period of 83 months (range: 11–108 months, interquartile range: 64–95 months), 59 patients (36.4%) developed cardiovascular events. The cardiovascular events included 17 (10.5%) coronary artery diseases requiring revascularization, 14 (8.6%) cerebrovascular accidents, nine (5.5%) hospitalization for heart failure, six (3.7%) pulmonary embolism, four (2.4%) aortic aneurysm and dissection, and nine (5.5%) development of sustained ventricular or supraventricular arrhythmias. Cox proportional HR analysis revealed few statistically significant associations between clinical and echocardiographic data of patients with SLE and the risk of cardiovascular events (Table 4). In the univariate Cox regression models, endocardial CS had a significant and relatively higher HR for cardiovascular events (HR, 2.508; 95% CI, 1.501–4.191; *P* < 0.001), as did age (HR, 1.045; 95% CI, 1.021–1.073; *P* = 0.031), systolic blood pressure (HR, 1.021; 95% CI, 1.005–1.038; *P* = 0.010), SLEDAI-2000 score (HR, 1.030; 95% CI, 1.011–1.050; *P* = 0.008), sPAP (HR, 1.064; 95% CI, 1.041–1.088; *P* < 0.001), and endocardial LS (HR, 1.055; 95% CI, 1.031–1.156; *P* < 0.001). On the contrary, positive antiphospholipid antibodies, IVSd, LVEF, LVM, LS gradient, and LS gradient were not significantly associated with cardiovascular events. In multivariate Cox regression analysis, factors were chosen on the basis of their significance in univariate analysis; in addition to those, the following additional factors were included in the model: age, systolic blood pressure, SLEDAI-2000 score, sPAP, and LV endocardial LS and CS. In this model LV endocardial LS(HR, 1.014; 95% CI, 1.002–1.035; *P* = 0.025) and endocardial CS (HR, 1.051; 95% CI, 1.027–1.077; *P* < 0.001) were independent association with cardiovascular events.

**Table 4:** Cox regression uni- and multivariate analyses for the occurrence of cardiovascular events in patients with SLE

Clinical and echocardiographic parameter	Univariate analysis		Multivariate analysis	
	**HR(95% CI)**	***P*-value**	**HR(95% CI)**	***P*-value**
Age	1.045 (1.021–1.073)	**0.031**	1.160 (0.339–3.375)	0.471
Sex, female	0.728 (0.264–2.011)	0.540		
Systolic blood pressure	1.021 (1.005–1.038)	**0.010**	0.984 (0.959–1.089)	0.223
Diabetes mellitus	1.320 (0.781–2.170)	0.312		
SLEDAI-2000 score	1.030 (1.011–1.050)	**0.008**	1.019 (0.994–1.044)	0.162
Positive anti-double-stranded DNA	1.356 (0.813–2.261)	0.244		
Positive antiphospholipid antibodies	1.396(0.765–2.548)	0.277		
Glucocorticoids	0.956 (0.565–1.618)	0.868		
IVSd	0.983 (0.799–1.208)	0.868		
LVEDd	0.997 (0.943–1.055)	0.926		
EDV	1.006 (0.991–1.021)	0.462		
LVEF	0.962 (0.910–1.018)	0.962		
LVM	1.001 (0.991–1.021)	0.802		
E/ e′	0.973 (0.784–1.207)	0.801		
sPAP	1.064 (1.041–1.088)	** < 0.001**	1.018 (0.848–1.431)	0.056
Endocardial LS	1.055 (1.031–1.156)	** < 0.001**	1.014 (1.002–1.035)	**0.025**
LS gradient	1.076 (0.701–1.645)	0.738		
Endocardial CS	2.508 (1.501–4.191)	** < 0.001**	1.051 (1.027–1.077)	** < 0.001**
CS gradient	1.114 (0.811–1.529)	0.505		

***NOTE:**** HR* hazard ratios SLEDAI-2000 score, *SLE* disease activity index-2000 score, *IVSd* interventricular septum diastolic, *LVEDd* left ventricular end-diastolic diameter; *EDV* end-diastolic volume, *LVEF* left ventricular ejection fraction, *LVM* left ventricular mass, *LVMI* left ventricular mass index, *LAVI* left atrial volume index, *E* early-diastolic velocity and the average values of early-diastolic velocities at the septal and lateral mitral annulus ratio, *sPAP* systolic pulmonary artery pressure, *LS* longitudinal strain, *CS* circumferential strain

**Fig. 4:**
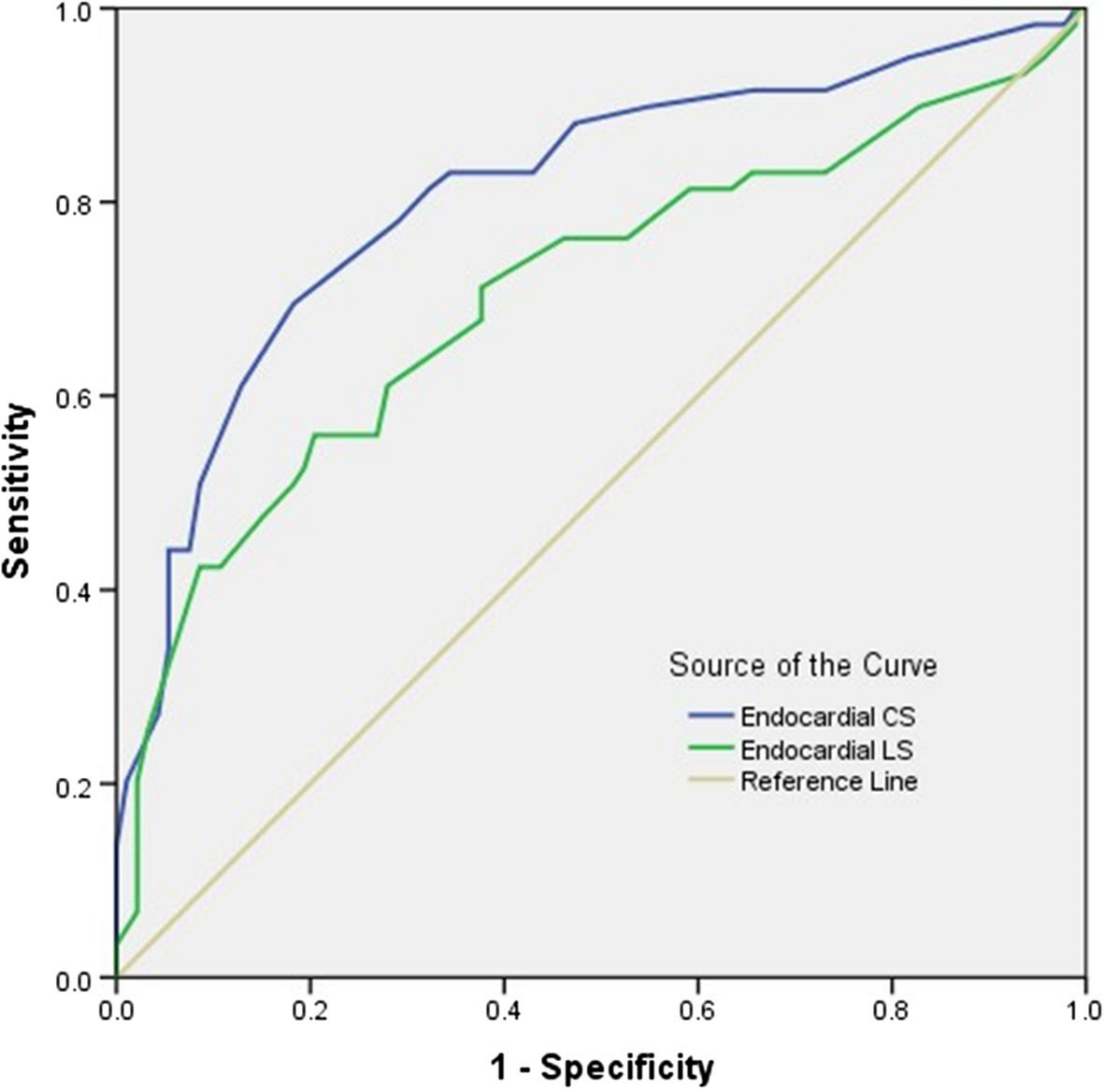
Receiver operating characteristic curve analysis for the prediction of cardiovascular events in patients with SLE. The cutoff values of LV endocardial LS and endocardial CS were -21.5% (sensitivity: 75.6%, specificity: 64.0%) and -29.0% (sensitivity: 82.2%, specificity: 74.9%) with areas under the curves of 0.746 and 0.829, respectively

**Fig. 5:**
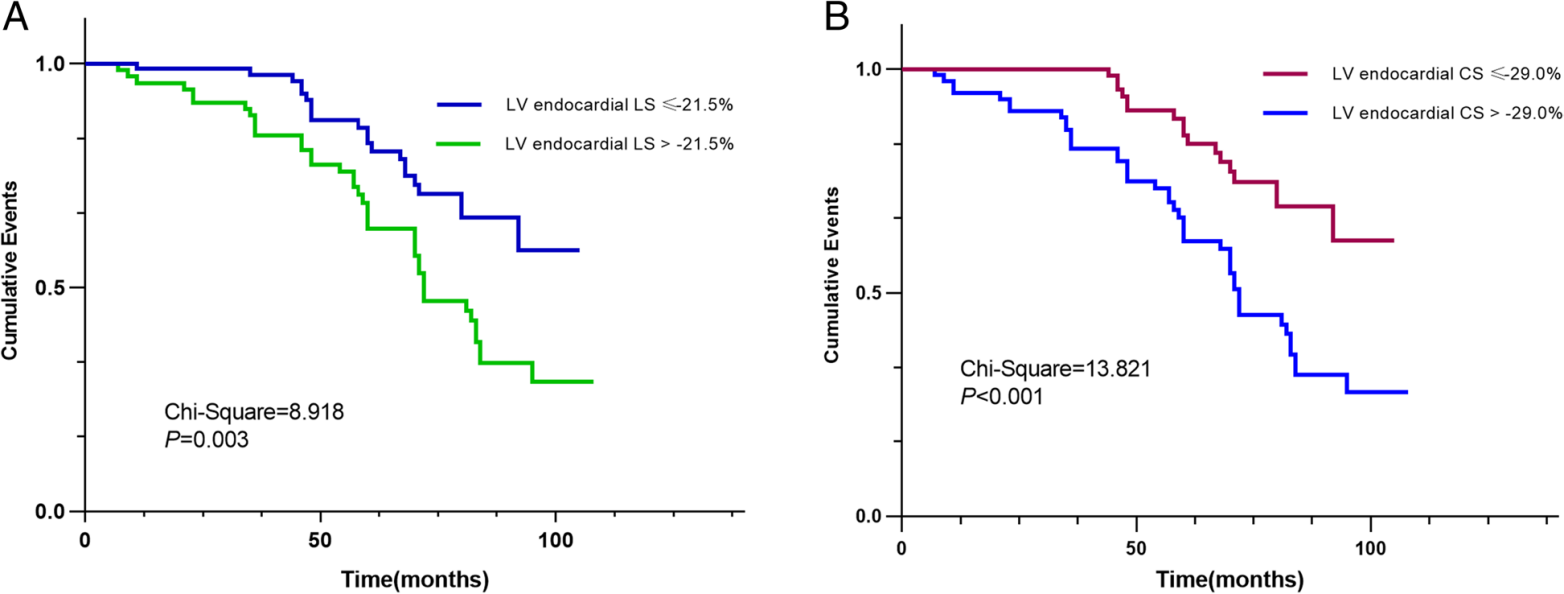
Kaplan–Meier curves showing the association of LV endocardial LS (A: dichotomized according to the value of -21.5%) and endocardial CS (B: dichotomized according to the value of -29.0%) with the development of cardiovascular events

In addition, the results of the ROC analyses are presented in Table 5 and Fig. 4. In the prediction of cardiovascular events, the cutoff values of endocardial LS and endocardial CS were -21.5% (sensitivity: 75.6%, specificity: 64.0%) and -29.0% (sensitivity: 82.2%, specificity: 74.9%), with areas under the curves of 0.746 and 0.829, respectively. Patients with SLE were divided into two groups based on cutoff values of LV endocardial LS or endocardial CS. The Kaplan–Meier log-rank test revealed significant differences in the overall cumulative rates of cardiovascular events in both separations. Patients with SLE with more impaired LV endocardial LS had higher cumulative rates of cardiovascular events compared to patients with less impaired LV endocardial LS ≤ -21.5% (χ^2^ = 8.918, *P* = 0.003); meanwhile, patients with lower LV endocardial CS had higher cumulative rates of cardiovascular events compared to patients with an absolute higher value of endocardial CS ≥ 29.0%(χ^2^ = 13.821, *P* < 0.001, Fig. 5).

**Table 5:** Receiver operating characteristic curve analysis for predictions of cardiovascular events in patients with SLE

Parameter	Cardiovascular events			
	**AUC**	**Sensitivity (%)**	**Specificity (%)**	**Cutoff point**
Endocardial LS	0.746	75.6	64.0	-21.5 (%)
Mid-myocardial LS	0.677	65.3	63.6	-18.1 (%)
Epicardial LS	0.612	61.0	64.2	-17.1 (%)
LS gradient	0.705	70.2	63.4	-3.3 (%)
Endocardial CS	0.829	82.2	74.9	-29.0 (%)
Mid-myocardial CS	0.725	75.4	66.8	-18.0 (%)
Epicardial CS	0.623	58.4	70.6	-12.2 (%)
CS gradient	0.715	70.8	65.4	-17.1 (%)

***NOTE:**** AUC* area under the curve, *LS* longitudinal strain, *CS* circumferential strain

### Reproducibility

The ICC for intra- and inter-observer reproducibility was 0.95 and 0.89 for LV endocardial LS, 0.94 and 0.91 for endocardial CS, 0.92 and 0.90 for mid-myocardial LS, 0.89 and 0.87 for mid-myocardial CS, 0.88 and 0.86 for epicardial LS, and 0.87 and 0.85 for epicardial CS.

## Discussion

In this study, we found that (1) LV systolic function in patients with SLE was significantly lower compared to healthy control subjects on routine echocardiography and LSS technology; (2) in these patients, LV endocardial LS and CS were independently associated with the occurrence of cardiovascular events, whereas conventional echocardiographic parameters of LV structure and systolic function were not.

### LV systolic function in patients with SLE

SLE is one of the strongest risk factors for CVD [1, 2], which can result in many forms of cardiovascular involvement, including pericarditis, valvular disease, myocardial fibrosis, inflammatory, myocarditis, atherosclerotic or thromboembolic change in the artery, coronary artery disease, congestive heart failure, arrhythmias, conduction abnormalities, cerebrovascular accident, pulmonary embolism, and aortic aneurysm and dissection [14, 15]. The diagnosis of lupus myocarditis is often based on clinical grounds after evaluation by coronary angiography and other cardiac imaging procedures; however, myocardial involvement at an early stage is still under-diagnosed because of the subtle and nonspecific clinical manifestations and the limitations of current clinical diagnostic technology. Clinically evident lupus myocarditis is identified in less than 10% of patients with SLE [16, 17]; however, it is noted in up to 70% of patients at autopsy as well as antemortem on echocardiography [3, 18]. In this study, we found changes in conventional echocardiographic parameters involved in LV structure and functional abnormalities, including an increase in IVSd, LVDd, EDV, and LVMI and a decrease in LVEF, E, e’, and E/e’ diastolic function of the LV in patients with SLE. However, these parameters were in the normal range with a low prevalence of comorbidities in patients with SLE without heart failure symptoms. Studies have also shown that speckle-tracking echocardiographic parameters compared to the LVEF is a good predictor of mortality in patients with SLE without heart failure symptoms [19, 20]. The results of the LSS assessment showed a significant decrease in LV three-layer LS and CS and transmural strain gradient in patients with SLE compared to the healthy controls. Furthermore, when assessing LV systolic function with more sensitive tools such as endocardial LS and CS were significantly lower than in control subjects in a certain percentage of patients was substantially impaired according to the standard values proposed by a current study [21, 22]. These findings suggest that subclinical impairment of LV function can occur even when LVEF remains in the normal range in patients with SLE. A previous study has shown an impairment of LV endocardial LS in 35 adult patients with SLE compared to healthy controls [23], but not LV endocardial CS. Compared with this study, it may exclude patients with a history of drug treatment and cardiovascular disease-related factors. Cumulative evidence strongly supports traditional, disease-related risk factors and medications of treatment can lead to lupus myocarditis [24]; evidence shows that CS and LS play different roles in LV systolic function, with circumferential shortening contributing more to LVEF, LS decreases in the early stage of impaired myocardial function [25].

### Clinical and echocardiographic associates of cardiovascular events in SLE

Patients with SLE have up to a 10- fold risk of cardiovascular morbidity and mortality compared to the general population, and cardiac involvement still accounts for most of the deaths [1, 2]. Previous studies have identified clinical and ultrasonic medical measures prognostic factors in patients with SLE; however, few of them focused on cardiovascular events, and no studies so far have explored the predictive value of LSS technique measures such as LV endocardial LS and CS. The incidence of cardiovascular events in this study was 36.4%, inconsistent with previous reports that the occurrence of cardiovascular events varies from 15 to 55% in SLE patients [17, 26]. Meta-analyses and systematic reviews demonstrated that the activity and severity of the disease have a small prognostic impact on cardiovascular events [6, 27, 28]. In this study, clinical data such as age, systolic blood pressure, and SLEDAI-2000 score were significantly associated with the occurrence of cardiovascular events, while positive anti-double-stranded DNA, positive antiphospholipid antibodies, and drugs for the treatment of SLE were not significantly associated with outcome. On the contrary, in the multivariate analysis, age, systolic blood pressure, and SLEDAI-2000 score did not show significant associations with the occurrence of cardiovascular events. The limited prognostic value of the SLEDAI-2000 scores observed in this study might be explained by the fact that some important alterations are not considered, and the SLEDAI-2000 score does not obtain the activity and severity of the disease with an organ system, such as cardiopulmonary manifestations, hemolytic anemia, and myositis.

In this study, conventional echocardiographic and LSS technique parameters such as sPAP, LV endocardial LS, and endocardial CS were significantly associated with cardiovascular events in univariate analysis, while mid-myocardial and epicardial strains and transmural strain gradient were not significantly associated with outcome. Only LV endocardial LS and CS were significantly associated with cardiovascular events in multivariate analysis. In addition, the results of ROC analysis revealed the statistical significance of LSS technique parameters in the prediction of cardiovascular events. LV endocardial LS and CS were more powerful and had better predictive power; LV endocardial CS had the highest HR and the most prominent areas under the curves. Therefore, LV endocardial strains may be able to reflect not only subclinical myocardial involvement at an early stage but also an overall increase in risk for cardiovascular events in patients with SLE. Even when LV systolic function seems normal under conventional echocardiographic parameters, micro- and macrovascular abnormalities might affect LV endocardial strains and can lead to cardiovascular events. Recently, the association of subclinical LV myocardial dysfunction by layer-specific LS could better predict significant stenosis in the coronary artery supplying the surrounding territory in patients with suspected stable angina pectoralis and normal LV wall motion [29]. In a long-term follow-up study in patients with acute myocardial infarction, endocardial strains had better predictive power for the occurrence of cardiovascular events after myocardial infarction, which indicates that evaluation of myocardial damage at the endocardial layer is crucial in clinical management and outcomes [30].

LV endocardial LS and CS could be considered as a new tool to diagnose subclinical (primary) myocardial involvement and to optimize individual patient management and risk stratification.

### Study limitations

There are some limitations to this study that should be acknowledged. In this study, we enrolled SLE patients with CVD-related risk factors (including smoking, hypertension, diabetes mellitus, dyslipidemia, obesity, etc.); therefore, the results cannot be applied to an SLE population without CVD-related risk factors. We could not exclude the possibility that pulmonary hypertension contributes to impaired LV myocardial function in patients with SLE. Further study should be conducted to include a larger cohort of patients in a multicenter setting to clarify these results.

## Conclusions

To the best of our knowledge, this is the first study to explore the association between LSS technique parameters and the development of cardiovascular events in patients with SLE. LV systolic function is significantly lower than healthy control subjects on routine echocardiography and LSS technology. The LV endocardial LS and CS have better than the other two layers strain predictive value for cardiovascular events, which indicates especial endocardial is crucial for clinical management and may significantly improve risk stratification in the early evaluation of early evaluation myocardial involvement for patients with SLE.

## Data Availability

The data and material in the current study are available from the corresponding author on reasonable request.
